# Survival benefits of interventional radiology and surgical teams collaboration during primary trauma surveys: a single-centre retrospective cohort study

**DOI:** 10.1186/s12873-024-00977-0

**Published:** 2024-04-16

**Authors:** Ichiro Okada, Toru Hifumi, Hisashi Yoneyama, Kazushige Inoue, Satoshi Seki, Ippei Jimbo, Hiroaki Takada, Koichi Nagasawa, Saiko Kohara, Tsuyoshi Hishikawa, Hiroki Shiojima, Eiju Hasegawa, Kohei Morimoto, Yoshiaki Ichinose, Fumie Sato, Nobuaki Kiriu, Junichi Matsumoto, Shoji Yokobori

**Affiliations:** 1 Department of Emergency and Critical Care Medicine, Nippon Medical School Hospital, 1-1-5, Sendagi, Bunkyo-ku, 113-8603 Tokyo, Japan; 2 Department of Critical Care Medicine and Trauma, National Hospital Organization Disaster Medical Center, 3256, 190-0014 Midoricho, Tachikawa city, Tokyo, Japan; 3 Department of Emergency Medicine, St. Luke’s International Hospital, 9-1, Akashicho, Chuo-ku, 104-8560 Tokyo, Japan; 4 Department of Anesthesia, Kyorin University Hospital, 6-20-2 Shinkawa, 181-8611 Mitaka city, Tokyo, Japan; 5 Department of Radiology, Kawasaki Municipal Tama Hospital, 1-30-37 Shukugawara, Tama-ku, 214-8525 Kawasaki city, Japan; 6 Department of Radiology, National Hospital Organization Disaster Medical Center, 3256, 190-0014 Midoricho, Tachikawa city, Tokyo, Japan; 7 Department of Traumatology and Critical Care Medicine, National Defense Medical College, 3-2, 359-8513 Namiki, Tokorozawa city, Saitama Japan; 8 Department of Emergency and Critical Care Medicine, St Marianna University School of Medicine, 2-16-1, Sugao, Miyamae-ku, 216-8511 Kawasaki city, Japan

**Keywords:** Damage control interventional radiology, Damage control surgery, Time process, Transcatheter arterial embolization, Treatment selection

## Abstract

**Background:**

A team approach is essential for effective trauma management. Close collaboration between interventional radiologists and surgeons during the initial management of trauma patients is important for prompt and accurate trauma care. This study aimed to determine whether trauma patients benefit from close collaboration between interventional radiology (IR) and surgical teams during the primary trauma survey.

**Methods:**

A retrospective observational study was conducted between 2014 and 2021 at a single institution. Patients were assigned to an embolization group (EG), a surgery group (SG), or a combination group (CG) according to their treatment. The primary and secondary outcomes were survival at hospital discharge compared with the probability of survival (Ps) and the time course of treatment.

**Results:**

The analysis included 197 patients, consisting of 135 men and 62 women, with a median age of 56 [IQR, 38–72] years and an injury severity score of 20 [10–29]. The EG, SG, and CG included 114, 48, and 35 patients, respectively. Differences in organ injury patterns were observed between the three groups. In-hospital survival rates in all three groups were higher than the Ps. In particular, the survival rate in the CG was 15.5% higher than the Ps (95% CI: 7.5–23.6%; *p* < 0.001). In the CG, the median time for starting the initial procedure was 53 [37–79] min and the procedure times for IR and surgery were 48 [29–72] min and 63 [35–94] min, respectively. Those times were significantly shorter among three groups.

**Conclusion:**

Close collaboration between IR and surgical teams, including the primary survey, improves the survival of severe trauma patients who require both IR procedures and surgeries by improving appropriate treatment selection and reducing the time process.

## Background

A team approach, in which relevant divisions collaborate beyond their boundaries, is essential for effective management of trauma cases [1]. Interventional radiology (IR) procedures for patients with torso trauma have been widely accepted as effective hemostatic measures [2], even among patients with potentially lethal multiple injuries [3]. Consequently, a close collaboration between interventional radiologists and surgeons during the initial management of trauma patients is important for prompt and accurate care.

Recently, an approach in which an IR team comprising radiologists who participate in the entire treatment process, especially in the primary survey of trauma patients, has been advocated [4]. This approach is expected to shorten the treatment process and improve outcomes, particularly in severe trauma patients. A recent study demonstrated that participation of an IR team in the primary survey (PS) of hemodynamically unstable patients with torso trauma improved their survival by 24.6% compared with the predictive survival rate [5].

However, the types of trauma patients who would benefit from a close collaboration between interventional radiologists and surgeons in the PS remain unclear. Thus, we aimed to determine whether patients receiving any type of treatment would benefit, in terms of survival, from a collaboration between IR and surgical teams.

## Methods

### Study design

This retrospective cohort study was conducted at the National Hospital Organization Disaster Medical Center, Tokyo, Japan. The medical records of all patients who sustained trauma and were admitted to the intensive care unit at our center between January 2014 and December 2021 were examined.

### Patient population

Patients with an abbreviated injury score (AIS) of ≥ 3 for the torso who underwent transcatheter arterial embolization (TAE) or surgeries were enrolled in the study. The exclusion criteria included cardiopulmonary arrest upon hospital arrival, TAEs or surgeries performed > 8 h after hospital arrival, age < 15 years, or withdrawal of treatment. Patients were grouped based on their treatment as follows: embolization group (EG), surgery group (SG), and combination group (CG). Patients in the EG only underwent TAEs during initial trauma management, and patients in the SG only underwent surgeries. Patients in the CG underwent both TAEs and surgeries during initial management. The patients were resuscitated according to the Japan Advanced Trauma Evaluation and Care guidelines [6].

### Interventions

**Fig. 1:**
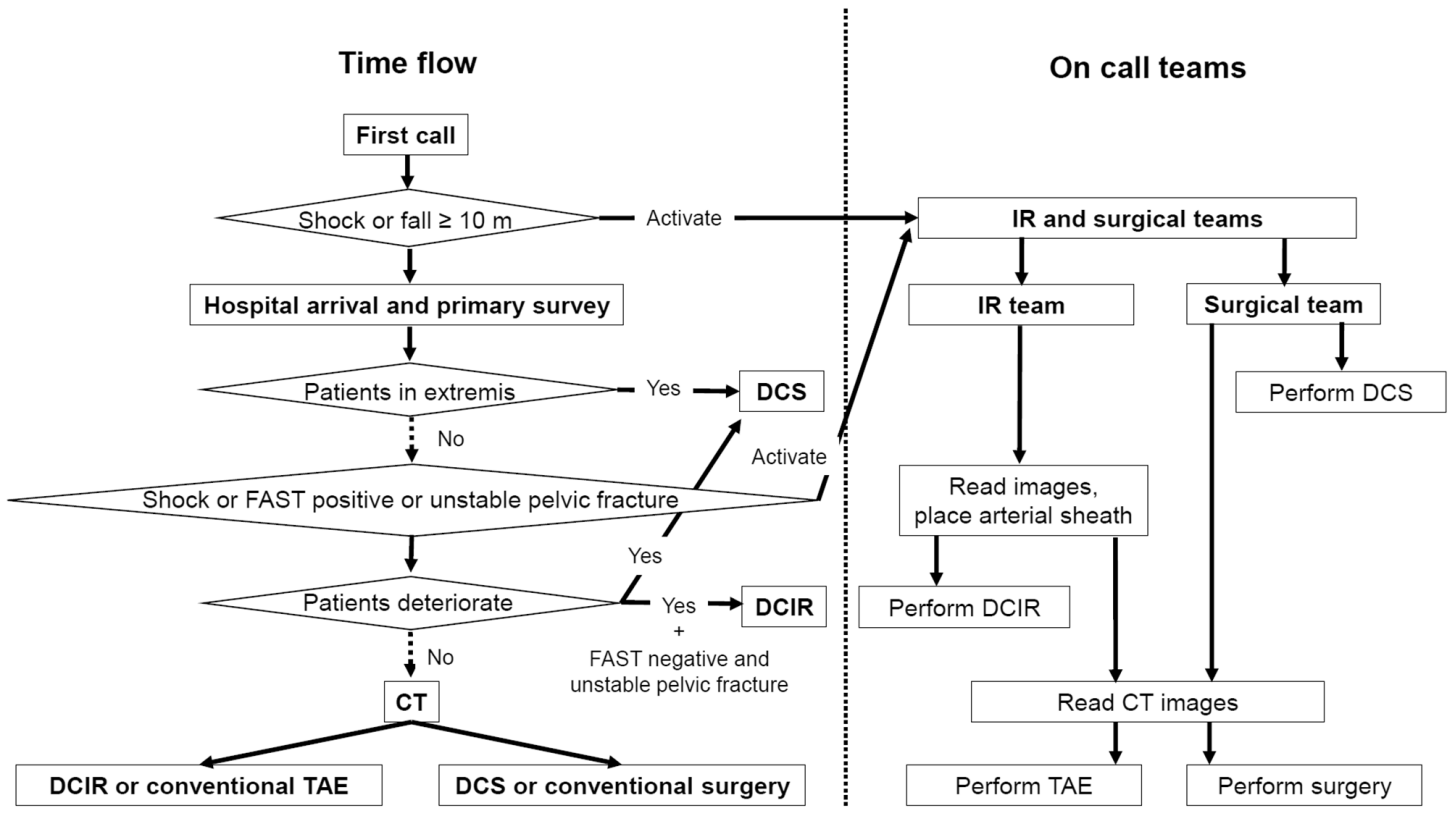
Process of initial trauma management. CT, computed tomography. DCIR, damage control interventional radiology. IR, interventional radiology. DCS, damage control surgery. FAST, focused assessment with sonography for trauma. IR, interventional radiology. TAE, transcatheter arterial embolization

The head of the emergency physicians was the team leader for initial trauma management. Both the IR and surgical teams, each consisting of at least two or more personnel, were on call 24/7. Each team included at least one board-certified doctor. Indications for IR procedures and surgeries were discussed among the leaders of the emergency physicians, IR, and surgical team before performing the procedures. Both the IR and surgical teams were alerted when a first call was received from the prehospital emergency medical service regarding a patient in shock following a trauma or a patient with a history of fall from height of ≥ 10 m. Damage control surgeries were performed when patients were in extremis. The IR team participated in the diagnostic evaluation of the patients presenting following a trauma by reading images, including focused assessment with sonography for trauma, X-ray, and computed tomography (CT). When TAEs were expected to be required, the IR team placed an arterial sheath for IR procedures. If patients deteriorated, damage control surgeries or damage control IR procedures were performed [4]. Damage control IR was defined as a time-conscious IR strategy to establish hemostasis as quickly as possible, at least within 1 h. CT was performed in two phases (i.e., arterial and parenchymal) according to the institutional trauma CT protocol from the head to the pelvis using an iodinated contrast agent. During patient resuscitation, indications for interventions were decided on the basis of a comprehensive evaluation of the patient’s characteristics, hemodynamic status, and imaging. The IR team performed the procedures with support from the stand-by surgical team. Conversely, the surgical team performed the surgeries with the support from the IR team. If hemostasis could not be achieved within 1 h, switching to surgery or IR was considered. When switching to surgery or IR, the patient was immediately transported to the operating theater or angiography suite. The trauma bay, CT room, and angiography suite are separate and located 30 m apart from each other, and the operating theater is located on the second floor. The initial management process is shown in Fig. 1.

### Study outcomes

The primary outcome of in-hospital survival was compared with the probability of survival (Ps). Secondary outcomes included the time to start the procedure, procedure time, time to switch to another procedure, and complications.

The Ps was calculated using the trauma and injury severity score (TRISS) methodology [7]. The difference between the observed in-hospital survival and Ps was determined using the following equation: TRAIS (TRISS adjusted increment in survivability) = 1 (alive) or 0 (dead)–TRISS Ps [8].　The time to start the procedure was defined as the duration between hospital arrival and skin puncture, skin incision, or catheter insertion when the arterial sheath was placed at the trauma bay. The time to switch to another procedure was defined as the duration between the end of the former procedure and the start of the next procedure. “Shock” was defined as a systolic blood pressure of < 90 mmHg during the PS [9]. “Complications” were defined as any adverse event that required surgical, endoscopic, or radiological intervention, with a Grade ≥ 3 according to the Clavien–Dindo classification [10].

### Statistical analyses

Continuous variables are presented as medians and interquartile ranges. A one-sample Student’s t-test was used to analyze TRAIS. Other continuous variables were analyzed using the Kruskal–Wallis test for the comparison of three groups and Mann–Whitney test for the comparison of two groups. Categorical variables were compared using the Fischer’s exact test. A p-value of < 0.05 was considered statistically significant. The EZR (Saitama Medical Center, Jichi Medical University, Saitama, Japan) graphical user interface for R (The R Foundation for Statistical Computing, Vienna, Austria) was used to perform all statistical analyses [11].

## Results

### Patient characteristics

**Fig. 2:**
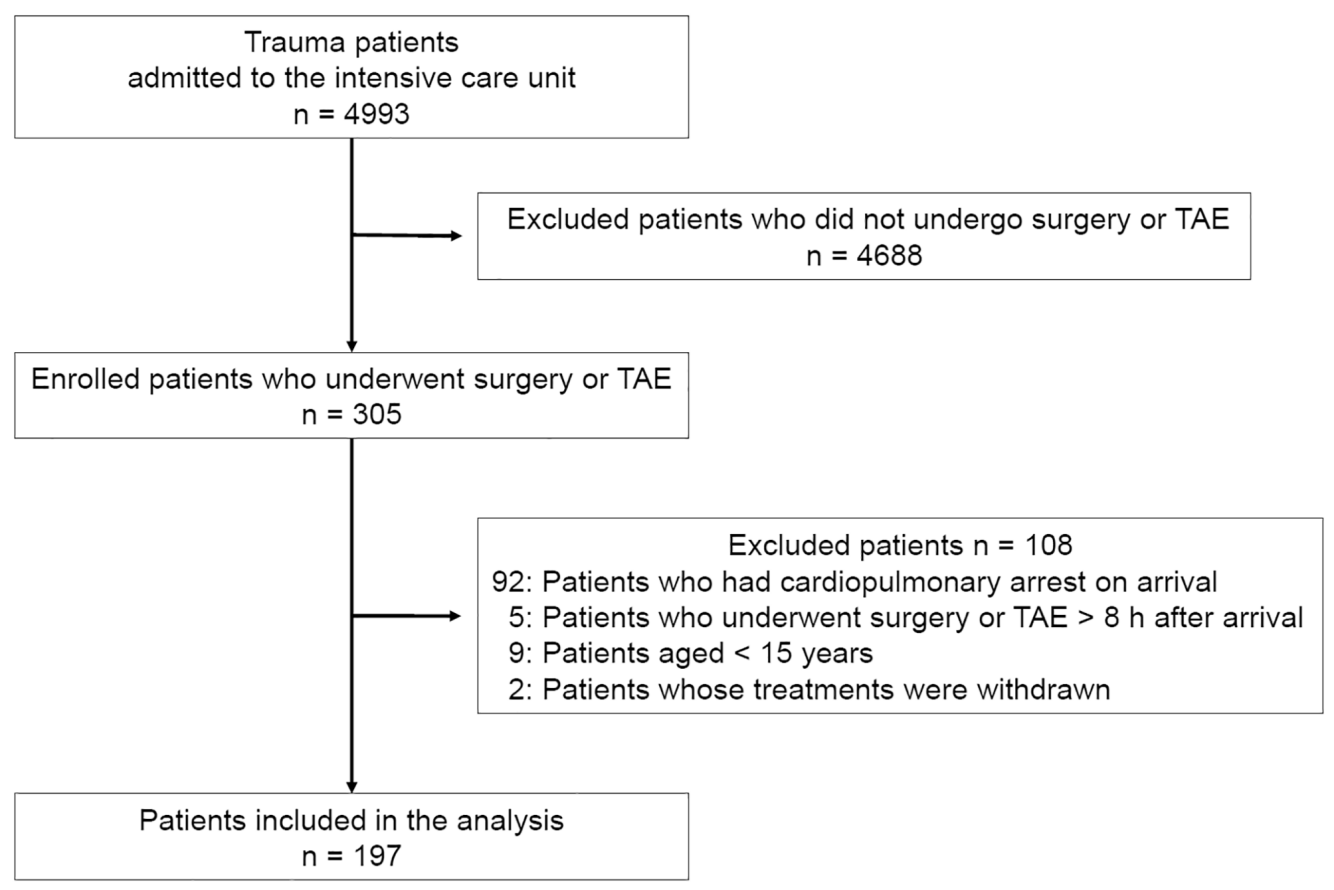
Flow diagram of study enrollment. TAE, transcatheter arterial embolization

During the study period, 4993 patients with a history of trauma were admitted to the intensive care unit. Of the 4993 patients, 305 had an AIS of ≥ 3 for torso injuries and underwent TAEs or surgeries. After excluding 108 patients, data from the remaining 197 patients (135 men and 62 women) were analyzed (Fig. 2).

The median age of the study population was 56 years [IQR: 38–72]. Twenty-seven patients suffered from a penetrating trauma and 170 patients suffered from a blunt trauma. The median ISS score was 20 [10–29], the median revised trauma score (RTS) was 7.84 [6.90–7.84], and the median Ps was 0.94 [0.85–0.98]. Shock developed in 59 patients during the PS. The EG, SG, and CG included 114, 48, and 35 patients, respectively. Patient data are shown in Table 1. The age, male ratio, injury mechanism, ISS, RTS, and Ps were significantly different between the three groups. The SG included more patients with penetrating injuries, and the CG had higher ISS, lower RTS, and lower Ps. Differences in organ injury patterns were observed among the three groups. No patients in the EG had an AIS of ≥ 3 for bowel and diaphragm injuries. No patient in the SG had an AIS of ≥ 3 for pelvis and spleen injuries, and no patient in the CG had an AIS of ≥ 3 for diaphragm injuries.

**Table 1:** Patient characteristics

	Embolization group (*n* = 114)	Surgery group (*n* = 48)	Combination group (*n* = 35)		P-value
Age (years)	64 [41–78]	47 [35–65]	43 [24–64]	< 0.001	
Male sex	70 (61.4)	39 (81.3)	26 (74.3)	0.038	
Blunt injury	110 (96.5)	30 (62.5)	30 (85.7)	< 0.001	
Shock	24 (21.1)	12 (25)	23 (65.7)	< 0.001	
Injury severity score	20 [13–29]	13.5 [9–21]	30 [17–41]	< 0.001	
Revised trauma score	7.84 [7.11–7.84]	7.84 [7.06–7.84]	7.11 [5.97–7.84]	0.008	
Probability of survival	0.94 [0.85–0.97]	0.97 [0.93–0.99]	0.91 [0.67–0.96]	0.002	
Torso injury (AIS of ≥ 3)					
Pelvis Rib cage Liver Lung Bowel Spleen Spine Kidney Aorta Vascular of neck Mesentery Diaphragm Others	51 (44.3) 36 (31.6) 20 (17.5) 19 (16.7) 0 17 (14.9) 12 (10.5) 11 (9.6) 4 (3.5) 2 (1.8) 1 (0.9) 0 15 (13.2)	0 6 (12.5) 7 (14.6) 6 (12.5) 22 (45.8) 0 1 (2.1) 1 (2.1) 2 (4.2) 7 (14.6) 4 (8.3) 5 (10.4) 6 (12.5)	23 (65.7) 11 (31.4) 7 (20) 9 (25.7) 3 (8.6) 7 (20) 6 (17.1) 3 (8.6) 4 (11.4) 1 (2.9) 4 (11.4) 0 7 (20)	< 0.001 0.029 0.81 0.281 < 0.001 0.002 0.049 0.237 0.202 0.004 0.004 < 0.001 0.541	

AIS, abbreviated injury score

Data are expressed as numbers (percentages) or medians [interquartile ranges]

### Interventions

TAE was performed at 209 sites in 149 patients. TAEs involved the pelvis in 75 patients (75/149; 50.3%), the lumbar arteries in 25 patients (25/149; 16.8%), and the spleen in 22 patients (22/149; 14.8%). TAE was performed simultaneously at ≥ 2 sites in 44 patients (44/149; 29.5%). In the CG, 10 patients underwent TAE at ≥ 2 sites (10/35; 28.6%). Eighty-three patients underwent 125 surgical procedures. Bowel resection was the most frequently performed procedure (18/83; 21.7%), followed by hemostasis in the abdominal cavity (15/83; 18.1%), and bowel suturing (15/83; 18.1%). The median blood transfusion volume was 4 units [0–10] of red blood cells, 4 units [0–10] of fresh frozen plasma, and 0 units [0–0] of platelet concentrate. The interventions are shown in Table 2.

**Table 2:** Therapeutic interventions

	Embolization group (*n* = 114)	Surgery group (*n* = 48)	Combination group (*n* = 35)
Interventional radiology Sites for embolization			
Pelvis	53 (46.4)		22 (62.9)
Lumbar artery	16 (14.0)		9 (25.7)
Spleen	16 (14.0)		6 (17.1)
Liver	16 (14.0)		4 (11.4)
Costal artery	10 (8.8)		0
Kidney	5 (4.4)		3 (8.6)
Neck	3 (2.6)		3 (8.6)
Others	38 (33.3)		5 (14.3)
Surgical procedures Bowel resection		16 (33.3)	2 (5.7)
Bowel suture		10 (20.8)	5 (14.3)
Hemostasis in abdominal cavity		10 (20.8)	5 (14.3)
External fixation of pelvis		0	13 (37.1)
Hemostasis of neck Diaphragm suture Hepatorraphy Aorta cross clump Splenectomy Nephrectomy Open cardiac massage Others		7 (14.6) 5 (10.4) 6 (12.5) 2 (4.2) 1 (2.1) 0 2 (4.2) 15 (31.3)	2 (5.7) 2 (5.7) 0 3 (8.6) 3 (8.6) 3 (8.6) 1 (2.8) 12 (34.3)
Transfusion of blood products (unit)			
Red blood cell	2 [0–6]	0 [0–6]	12 [8–22]
Fresh frozen plasma	2 [0–8]	0 [0–6]	16 [9–30]
Platelet concentrate	0 [0–0]	0 [0–0]	0 [0–20]

Data are expressed as numbers (percentages) or medians [interquartile ranges]

### Effects on survival

A total of 183 patients survived to discharge (183/197; 92.9%). The in-hospital survival rates were 93.0% in EG, 93.8% in SG, and 91.4% in CG. Four patients in the EG, two in the SG, and three in the CG with a Ps of < 0.5 survived to discharge. Three patients with a Ps of ≥ 0.5 died in EG. The overall in-hospital survival rate was 8.3% higher than the Ps (95% confidence interval: 5.5–11.2%; *p* < 0.001). The in-hospital survival rates in all three groups were higher than the Ps. The survival rate in the CG was 15.5% higher than the Ps (95% confidence interval: 7.5–23.6%; *p* < 0.001) (Table 3).

**Table 3:** Observational survival rates compared with predictive survival rates

	Difference	95% confidence interval	P-value
Overall (*n* = 197) Embolization group (*n* = 114)	0.083 0.068	0.055–0.112 0.030–0.106	< 0.001 < 0.001
Surgery group (*n* = 48) Combination group (*n* = 35)	0.067 0.155	0.025–0.109 0.075–0.236	0.003 < 0.001

### Time course

The median time to start the initial procedure (TAE or surgery) was significantly shorter in the CG (53 [37–79] min) compared with the times in the other two groups (77 min in the EG and 90.5 min in the SG; *p* < 0.001). In the CG, 28 patients underwent IR first, and seven patients underwent surgery first. The median IR procedure time in CG was significantly shorter than that in EG (48 vs. 59 min; *p* = 0.021). Similarly, the median surgical time in CG was significantly shorter than that in SG (63 vs. 100 min; *p* < 0.001). The median time to switch procedures was 47 [28–108] min. The time course data are presented in Table 4.

**Table 4:** Time course

(min)	Embolization group (*n* = 114)	Surgery group (*n* = 48)	Combination group (*n* = 35)	P-value
Time to start	77 [57–105]	90.5 [52–116]	53 [37–79]	< 0.001
IR procedure time	59 [46–88]		48 [29–72]	0.021
Surgical time		100 [74–125]	63 [35–94]	< 0.001
Time interval			47 [28–108]	

Data are expressed as medians [interquartile ranges]

### Complications

Complications, including death, developed in 36 patients (36/197; 18.3%). The complication rates were 16.7% (19/114), 12.5% (6/48), and 31.4% (11/35) in EG, SG, and CG, respectively. Excluding death, the most common complication was pneumonia (*n* = 8, 4 in EG, 1 in SG, and 3 in CG), followed by pseudoaneurysms (*n* = 5, 4 in EG and 1 in CG), abdominal abscesses (*n* = 3, 1 in SG and 2 in CG), cerebral infarctions (*n* = 3, 2 in EG and 1 in SG), and wound infections (*n* = 2, 1 in SG and 1 in CG). All pseudoaneurysms were re-embolized and resolved. All abdominal abscesses were punctured and resolved. All infected wounds were debrided. No deaths directly related to the complications of procedures were observed.

## Discussion

The study results indicate that a workflow that includes a close collaboration between the IR and surgical teams during initial trauma management improves outcomes in patients who have sustained severe trauma and require both IR procedures and surgeries. Collaboration between the IR and surgical teams provides appropriate treatment and shortens the time to hemostasis.

In this study, the patients in CG demonstrated a higher survival rate than the TRISS-derived Ps by 15.5%. There are only a few studies that have compared the observed survival rate with the Ps [5, 8, 12]. Although the TRISS methodology was developed in the late 1980s [7], Ps is reportedly correlated with the actual survival even at present [13–15]. The TRAIS value allocates a baseline Ps as a substitute to each patient, which can be self-controlled [8]. Consequently, analysis using the TRISS methodology was considered appropriate for this retrospective study in a single institute, with the same study period and same teams.

IR procedures and surgeries have both advantages and disadvantages. Sites can often be approached more easily using IR procedures than surgery, such as the retroperitoneum, and hemostasis of multiple sites can be achieved simultaneously using IR without making multiple skin incisions [2, 16]. However, mechanical repair of the intestinal tract and diaphragm is not possible using IR. Furthermore, surgery can ensure reliable hemostasis and organ repair for patients in extremis under the supervision of anesthesiologists. In hemodynamically unstable patients with liver or pelvic trauma, a combination of surgery and TAE, such as perihepatic packing and hepatic arterial embolization, external fixation of pelvic fracture and arterial embolization, or preperitoneal pelvic packing and arterial embolization, are widely accepted as effective hemorrhage control [17, 18]. Appropriate treatment selection based on the advantages and disadvantages of each procedure is crucial. In this study, patients in the EG did not exhibit bowel or diaphragm injuries, and patients in the SG did not exhibit pelvic fractures or splenic injuries. In addition, TAE was usually selected for hemostasis of the costal arteries, lumbar arteries, and spleen, and surgery was selected for hemostasis of the cervical and mesenteric vessels. IR and surgical specialists contribute to prompt and appropriate treatment selection during PS in trauma patients.

Shortening the time to hemostasis is imperative. The mortality rate of patients with abdominal trauma who require surgery increases by 1% every 3 min in the emergency department [19]. Time delays of > 10 min between arrival in the emergency room and arrival in the operating theater increase the mortality rate in hypotensive patients with gunshot wounds to the torso [20]. Shortening the time to hemostasis becomes more important to the patient’s prognosis as injury severity increases. The present study demonstrated that the initiation of hemostasis (53 min), the initial procedure time (48 min for TAEs and 63 min for surgeries), and the time to switch to another procedure (47 min) were short in the CG. Nevertheless, TAEs were performed at > 2 sites in 28.6% of the patients in the CG. The time from hospital arrival to initiation of hemostasis using IR procedures in hemodynamically unstable trauma patients was reportedly 94 min, 46 min, and 53 min in the studies by Bize et al., Olthof et al., and Otsuka et al., respectively [7, 9, 10]. Moreover, a higher number of embolized sites was associated with prolongation of the procedure [21]. Considering these study results, our shortened time course is significant. A previous report advocated completing embolization within 10 min for each targeted vessel when treating trauma patients with severe hypotension [4]. Furthermore, a trauma hybrid operating room, in which both IR procedures and surgeries can be performed simultaneously, is associated with earlier hemostasis, leading to fewer blood transfusions, infectious complications, and days on ventilator support [22]. Performing hemostasis promptly and rapidly is important for improving patient prognosis. Earlier hemostasis may have contributed to the improved patient outcomes in this study.

A previous study demonstrated that close collaboration between the IR and surgical teams in PS during initial trauma management improves the survival rate of hemodynamically unstable patients who require TAE [5]. Using a similar team approach, this study demonstrated that the survival rate of severe trauma patients who required both IR procedures and surgeries was better than the Ps. Organized team collaboration is the key to team approach. On-the-job training and regular simulation drills for initial trauma management were conducted to improve collaboration between the two teams. Emergency physicians and other healthcare personnel, such as nurses and radiology technicians, also participated in the simulation drills using case scenarios to explore problems and cooperate appropriately. Simulation drills with multidisciplinary participation improve communication skills within the team [23], which are maintained through repetition [24]. These initiatives resulted in a unified response from the frontline workers, an increased awareness of the need to reduce the time to hemostasis, and improvement in initial trauma management.

Kinoshita et al. demonstrated that a hybrid emergency room, which integrates the computed tomography scanning room, angiography suite, and operating theater, could improve the outcomes of trauma patients [25]. However, emphasis should be placed on the importance of cooperation and skills development among the staff. The present study demonstrates that reinforcement of collaboration between the IR and surgical teams improves patient outcomes. This workflow can be employed anywhere in the world, by adjusting human factors without developing new facilities.

The present study has several limitations. First, this study was a single-center retrospective study that included a small sample size. Consequently, this study is subject to bias. In addition, no control groups were included in the study. Second, discrepancies between TRISS- derived Ps and actual survival rate may occur in patients with very high or very low Ps or in older adults with comorbidities [26, 27]. Third, protocols for IR and surgical indications were not predetermined and could have led to treatment selection bias among operators. Fourth, “shock” was defined as a systolic blood pressure of < 90 mmHg during PS in this study. However, no widely accepted quantitative definition of shock has been established.

## Conclusion

Close collaboration between the IR and surgical teams during PS can improve the survival of severe trauma patients requiring both IR procedures and surgeries. This workflow ensures appropriate treatment according to each injury and shortens the time to hemostasis.

## Data Availability

The datasets used and analyzed during the current study are available from the corresponding author on reasonable request.

## Abbreviations

AIS: Abbreviated injury score
CG: Combination group
CT: Computed tomography
EG: Embolization group
ISS: Injury severity score
IR: Interventional radiology
PS: Primary survey
Ps: Probability of survival
RTS: Revised trauma score
SG: Surgery group
TAE: Transcatheter arterial embolization
TRAIS: Trauma and injury severity score adjusted increment in survivability
TRISS: Trauma and injury severity score
